# A Solution for the Shortage of Detection Dogs: A Detector Dog Center of Excellence and a Cooperative Breeding Program

**DOI:** 10.3389/fvets.2018.00284

**Published:** 2018-11-16

**Authors:** Eldin A. Leighton, Elizabeth Hare, Scott Thomas, L. Paul Waggoner, Cynthia M. Otto

**Affiliations:** ^1^Canine Genetic Services LLC, Watertown, CT, United States; ^2^Penn Vet Working Dog Center, School of Veterinary Medicine, University of Pennsylvania, Philadelphia, PA, United States; ^3^Dog Genetics LLC, Sunnyside, NY, United States; ^4^American Kennel Club Detection Dog Task Force, Raleigh, NC, United States; ^5^Canine Performance Sciences, Auburn University, Auburn, AL, United States; ^6^Department of Clinical Sciences and Advanced Medicine, School of Veterinary Medicine, University of Pennsylvania, Philadelphia, PA, United States

**Keywords:** detection dog, breeding, semen, cooperative breeding, center of excellence

## Abstract

Currently, demand for US-bred and born detector dogs exceeds available supply, while reliance on foreign-bred sources introduces many unnecessary and unwanted risks. With proper management of a domestic supply line, U.S. breeders can improve both health and behavior by applying scientific principles to breeding and raising of detector dogs. A cooperative national detector dog breeding and development program will mitigate the current shortage of domestic-bred dogs that meet the health and behavior standards required by government, military, and law enforcement agencies. To coordinate such a cooperative, we propose a Detector Dog Center of Excellence (DDCoE) led by representatives of academic canine science programs guided by an advisory board of stakeholders. As a non-governmental organization, the DDCoE will oversee selective breeding of dogs owned by breeders, purchase the resulting puppies, and its members will supervise puppy raising until dogs are of a suitable age to be purchased by government agencies or other working dog organizations. The DDCoE will serve as an approved vendor to facilitate the procurement process. Breeding decisions will be based on proven quantitative genetic methods implemented by a specialized database. A national working dog semen bank will ensure conservation of diverse genetic material and enhance selection response by providing numerous potential sires. As a data collection and genetic evaluation center, the DDCoE will lead research to define quantitative traits involved in odor detection, to understand how these traits develop, and methods to optimize training of dogs endowed with enhanced odor detection ability.

## Introduction

The increase in frequency of terrorist attacks and natural disasters in the U.S. over the last two decades and the increased understanding of canine olfaction and training have led to a greater demand for healthy detector dog candidates (1, 2). Despite the “Buy American Act” mandating that products for government use be purchased from domestic sources, the Federal government, historically, and vendors currently supplying diverse United States agencies import most of their working dogs. These dogs frequently come with limited medical and training history data, often inaccurate ages, and their success rates are highly variable. Recent US legislation (3, 4) has encouraged domestic breeding of detection dogs, and some legislators have recommended restricting procurement of government working dogs to domestic sources.

To be prepared to meet the demands for domestic working dogs in the U.S., two major hurdles need to be surmounted. First is navigation of the government procurement process. Domestic suppliers of goods, including dogs, to the US Federal government must meet the Federal Acquisition Regulation (5). Potential vendors have a narrow window of opportunity to submit a cost proposal, which if accepted and the vendor meets all purchase regulation requirements, authorizes the vendor to bid on Federal contracts. These criteria differ among Federal agencies based on the type of work, working environment, training practices, and reward system (toy or food) used by each agency. Individual dogs could be matched with organizations based on behavioral phenotypes, since purpose-bred litters often contain pups with a range of behaviors. In the future, precise definition of work-related behaviors and standardization among agencies would facilitate breeding, selection, raising, and training. This hurdle associated with procurement, along with more lucrative sporting markets has reduced the incentive for domestic breeders to supply working dogs. The second hurdle is to increase the incentives and support for domestic breeders to breed working dogs.

Over the past 50 years, U.S. Federal government agencies such as Transportation Security Administration (TSA) (6), Customs and Border Protection (CBP; https://www.cbp.gov/border-security/along-us-borders/canine-program), U.S. Customs Service, and the U.S. Army (7) have initiated breeding programs. In every case, each of these breeding programs was either disbanded or dramatically reduced due to funding cuts. Clearly, decision-makers did not understand that steady, long-term genetic improvement requires at least three generations of selective breeding based on experience of guide (8), service, and military canine breeding programs (9).

To address current issues with quality and availability of detection dogs in the US, a plan for creating and administering a canine breeding cooperative is described. This cooperative will coordinate organizations and breeders to produce healthy, high-quality purpose-bred scent detection dogs for distribution across agencies. A basic tenet of the plan is to keep ownership of the breeding stock in the hands of private breeders and organizations. Doing so will help ensure the long-term survival of genetically advanced breeding stock, especially during times of scarce government funding.

To execute this plan, a federally supported Detector Dog Center of Excellence (DDCoE) is needed to coordinate the breeding plan, provide oversight of puppy development, collect data for continued genetic and training improvement, and negotiate the many complex issues of US Government purchase orders. The DDCoE will be led by representatives of academic canine science programs focused on working dogs in U.S. veterinary schools. An advisory board will be comprised of stakeholders from Federal, state, and local government agencies, academic institutions, working dog training organizations, researchers, and breeders. One of this board's functions will be to balance the DDCoE's requirements to facilitate diverse participation while maintaining standards that will result in genetic improvement over generations.

Breeders and working dog breeding organizations will be invited to apply for membership in the breeding cooperative. DDCoE managers will screen both the people and the breeding bitches they are nominating for enrollment based on clearly defined standards. Because the DDCoE will serve as the product supplier, during times of reduced US government demand, the DDCoE will be able to sell dogs to state or local governments, working dog organizations, and individuals. This freedom will preserve the ability to remain fiscally solvent. Dogs in the DDCoE program that are unsuited for government scent detection work may be sold to agencies who will place them into other forms of detection or other service work. Ownership of puppies born to bitches enrolled in the program will be pre-determined by contractual agreement made before a bitch is bred. The DDCoE will assume responsibility for puppy raising and for phenotype measurements to identify the best dogs for replacement breeders and for the different working disciplines. When a dog is between 8 and 14 months old, it will enter the inventory of young adult dogs available for sale. The overall intent is to enable government and law enforcement agencies to buy American-bred working dogs selected for an innate scent detection ability, thus ensuring the nation a secure supply of healthy, well-socialized dogs working to maintain public safety, while providing a coordinated approach for the sale of these dogs.

The DDCoE will establish and adhere to ethical standards for the treatment of dogs in breeding and puppy raising activities. Selection of dogs and breeders will be made with the overall goal of producing dogs that are willing and enjoy their work, and will have long, healthy careers. Unethical treatment by breeders, raisers, or DDCoE personnel and volunteers will not be tolerated. Affiliated veterinary colleges and specialty trained veterinarians will provide high quality medical care. An adoption program for dogs not meeting selection criteria and retired dogs will be established. In the event that an alternative career cannot be found (e.g., as a service dog), the dog will be offered to puppy raisers or for placement as a sport or pet dog.

## Guidelines for the detection dog center of excellence

### Organization

- The DDCoE shall be a non-governmental organization so that it can be sustained for the decades required to establish and maintain a successful working dog breeding program. The network of cooperating private organizations will meet government vendor requirements and can sell dogs to the federal government, but at times of low government demand, dogs can also be sold to state and local governments, service dog organizations, dog sport enthusiasts, and pet homes.
- The DDCoE shall be a cooperative consisting of non-governmental working dog organizations sharing the goal of producing sufficient healthy, high-quality, purpose-bred dogs for scent detection work.
- Breeding stock shall be owned by these non-governmental organizations and private breeders to ensure that genetic improvement is not lost because a single breeding program is disbanded. A wide geographical distribution will prevent large losses from local disease outbreaks or disasters.
- Academic working dog centers located at veterinary schools, and other similarly interested institutions shall comprise the DDCoE governing board. This board will set goals for the breeding program and coordinate selective breeding decisions for individual dogs. Other stakeholders will serve on an advisory board.
- The DDCoE will be a 501(c)(3) non-profit or part of a non-profit so it can provide receipts for donations by private individuals, including donations of dogs.
- The DDCoE will be an aggregator of young dogs from working dog organizations, puppy raising programs, and breeders that meet the contract specifications of agencies such as the TSA.
- If the DDCoE model can work with academic working dog centers as the initial proof of concept, then the DDCoE Board of Governors will entertain the option of expanding institutional membership to include other veterinary schools and academic institutions with canine science programs and appropriate veterinary support. This would increase geographic diversity among owners of the breeding bitches. Furthermore, it would distribute some of the workload for supplying high-quality working dogs among a larger group of similar, but geographically separated schools and organizations.

### Breeding

- Semen Bank
  - To enable the use of genetically superior males as sires of puppies born into the DDCoE, a frozen semen bank must be established to augment natural service or fresh-chilled semen breeding. Proper use of frozen semen requires a network of veterinarians (theriogenologists) skilled in trans-cervical insemination (TCI), vaginal insemination, surgical insemination. and timely semen placement (10, 11).
  - Frozen semen on each stud collected shall be permanently stored in multiple geographic locations to prevent loss due to physical or natural disaster.
  - For optimal semen quality, collected semen must meet minimum standards for post-thaw motility, morphology and sperm count (12, 13).
- Puppies
  - Identification of Breeding Bitches
    1. Private breeders may nominate their bitches to participate for one or more litters.
    2. Bitches with superior characteristics that enter the DDCoE program may also be identified for breeding. In this case, “superior” characteristics will be determined by application of estimated breeding values combined into an overall selection index that emphasizes the traits that need the most improvement.
  - Each nominated bitch that meets defined health and behavior standards will receive extensive health screening and phenotypic evaluation from the DDCoE.
  - Acceptance of a bitch into the program means that the owner will have an opportunity to negotiate a litter ownership agreement with the DDCoE. The DDCoE has the right to refuse litters to ensure that demand is met, but not exceeded.
  - Coordination by the DDCoE will ensure that insemination, prenatal, and whelping care are provided for every bitch by qualified veterinarians, veterinary technicians, or by the dog's private owner, if that is their choice. Veterinarians or veterinary technicians may be affiliated with one of the participating veterinary schools, or with a private veterinary practice.
  - Before a mating is initiated, the bitch's owner will choose from several litter ownership options including
    1. Donation of the entire litter to the DDCoE,
    2. Sale of the entire litter to the DDCoE
    3. Owners could retain ownership of up to two puppies if seven or more puppies are weaned, or one puppy if between three and five puppies are weaned.
    4. The breeder could retain ownership of the litter and risks associated with selling the litter. DDCoE would have the first right of refusal to purchase as many weaned puppies as it requires.
  - Young breeding quality bitches identified among puppies born into the DDCoE program may be bred for up to two litters beginning on their first heat cycle after they pass 18 months of age, while continuing training. After whelping their second litter, each bitch will be ovariectomized and will enter the work force in an appropriate career path. Bitches will produce no more than two litters to help maintain genetic diversity and to keep the generation interval short. Genetic improvement occurs by a combination of generation turnover in concert with the application of selection pressure. By keeping the generation interval short, genetic change per year is maximized (14).
- Population Scaling

The number of matings required to produce 100 Labrador Retriever (LR) detector dogs can be scaled. The following guidelines can be used in the calculations:

- Assuming the best case scenario, conception rate is ~85%.
- Average litter size is 7.5 puppies. In a study on Norwegian Kennel Club registrations, mean litter size was 6.9 ± 0.2 for LR (15). In The Seeing Eye breeding program, mean litter size at birth for LR was 6.8 ± 2.3 (16). In one study of litter size using frozen semen, average litter size was 5.4 pups (11). These statistics describe the specific populations in which they were measured and are likely to vary for other populations such as LRs bred for detection work in the US. Mean litter size varies between breeds, so results will vary by breed. In the Norwegian study, German Shepherd Dogs and Belgian Shepherds (also known as Groenendael) had smaller litter sizes (6.1 ± 0.1 and 6.4 ± 0.4, respectively, and Golden Retrievers had larger litter sizes (7.5 ± 0.2) (15). In The Seeing Eye study, German Shepherd Dogs had a mean litter size of 6.4 ± 2.5 (16).
- Ninety four percent of puppies born will survive until weaning. In The Seeing Eye breeding program, mean litter size at weaning for LR was 6.4 ± 2.4 (16).
- Although early puppy screening at 8 weeks still remains a goal, many studies to date have failed to identify accurate predictors at this age (17–19). For the purpose of this calculation and until more accurate screening methods are developed, retention of 100% of the puppies is assumed.
- Ninety five percent of dogs will successfully complete the puppy raising phase.
- Eighty percent of dogs will pass medical screening at the end of the puppy raising phase.
- In the beginning, it is anticipated that between 30 and 50% of dogs will meet government contracting standards, but with the production of genetically improved puppies born into future generations, the success rate will improve. This estimated success rate is based on findings in working and guide dog programs and the experience of the authors. A study on the Swedish Dog Training Center (20), which trained dogs for patrol, detection, and guide work, reported that about 50% of the dogs selected for training were disqualified. A 30% success rate was reported for dogs entering advanced training at the South African Police Dog Center (21) and for dogs at the Tokyo Customs Canine Training Center (22). In work on predicting success in multiple guide and assistance dog programs, Duffy and Serpell (23) report the programs have training success rates between 30 and 50%. For Australian guide dog programs, success was reported as 50–56% (24). A survey study involving an international group of guide dog schools found success rates between 23 and 100% for dogs completing training and 13 to 100% for dogs still working 1 year after completing training (25).

Under these assumptions, 53 litters will need to be born in a year to produce 100 puppies that meet contracting requirements. This example may over or underestimate some of the factors influencing successful number of puppies. Data collection from the program is necessary to provide more accurate estimates. Some of the success rates will improve as the DDCoE refines processes for insemination, whelping, puppy raising and training, and as genetic improvement is made across the population over generations. The approach of early, flexible training that depends on each puppy's aptitudes is likely to significantly improve success rates as dogs can be prepared for careers in explosives (object or passenger screening) and narcotics detection, search and rescue (SAR), human remains detection (HRD), and other specialties (www.vet.upenn.edu/wdc). Although these fields of work all involve odor detection, different sets of behaviors are optimal for different settings. This flexibility is likely to allow for a higher success rate than working dog breeding programs that focus solely on a single criterion for a successful outcome. This approach requires clear definition and consistent scoring of phenotypes associated with olfaction ability and aspects of behavior to ensure that each dog is placed into its optimal working discipline (26).

### Puppy raising

- The DDCoE will have complete ownership of the puppies it purchases, enabling it to sell puppies to any government or private working dog organization. The DDCoE will be responsible for raising and socializing each puppy to maximize the probability that a young dog will meet government procurement requirements.
- The procurement process for each agency sets the price of a dog but does not require the government to purchase any minimum number of dogs. The DDCoE will be able to sell dogs not needed by agencies within the Federal government to state and local agencies, private working dog organizations (e.g., search and rescue), and private individuals.
- Assuming that puppies are born with a strong genetic foundation, then their socialization experience during their first year of life will largely determine each puppy's ultimate success or failure. To ensure proper socialization, DDCoE must utilize puppy raising protocols designed to meet the special needs of scent detection dogs, perhaps by adapting already existing protocols in use by academic working dog centers. Opportunities may exist to engage local college students to be puppy raisers, as well as other local residents within a reasonable driving time of one of the DDCoE member organizations. In some settings, it may be possible to use the 4-H youth program and other social agricultural infrastructures to recruit puppy raisers who could either be volunteers or paid a stipend. Alternatively, correctional institution-based detector dog programs may be recruited, expanded and replicated to provide a scalable solution to needed dog raising resources. This variety of puppy raising models may be necessary due to the difficulty of recruiting individuals willing to live with and provide basic training for dogs that will not stay with them. Data from all of the alternative raising strategies, including puppy raising professionals, will be collected to determine the most cost-effective methods to produce the highest success rates.

### Genetics

- The choice of a mate for a participating bitch will be made by the DDCoE using a data-driven quantitative genetics protocol set up by its governing board to select for priority traits.
- Organization of pedigree and phenotype data on a large number of dogs is needed to apply quantitative genetic selection methods to the complex traits important for working dogs. The International Working Dog Registry (IWDR; https://www.iwdr.org), newly developed by the International Working Dog Breeding Association (https://www.iwdba.org), provides a database with specialized features for storing canine pedigree, phenotype, and in future, genotype data.
- The IWDR calculates inbreeding coefficients for dogs and their potential offspring, providing a tool for minimizing the accumulation of inbreeding in the working dog population. Including inbreeding information in mating decisions will preserve genetic variability in the population and avoid deleterious health and reproductive effects associated with increases in inbreeding (27–30).
- The IWDR will be able to fit quantitative genetics models to phenotype data. The potential for improving quantitative traits can be assessed in part by calculating the heritability of the trait (31). These models can also provide estimated breeding values (EBV) for each dog for each trait. An EBV depends on the phenotype of the dog and of its relatives, with close relatives contributing more information to the value than more distant relatives. EBVs can be used to select the individuals most likely to pass on favorable alleles for complex traits to their offspring (32). This method has been used to improve mean hip joint conformation score in The Seeing Eye dog population (8), and for distraction index and search-related behaviors in the TSA breeding program (unpublished data). Although the heritability of behavior traits is often considered to be low, moderate heritabilities have been found for some working dog behaviors. A study on German Shepherd Dogs (GSD) and Labrador Retrievers (LR) at the Swedish Dog Training Center (SDTC) found heritabilities above 0.25 for courage (GSD: 0.26 ± 0.06; LR 0.28 ± 09), prey drive (GSD: 0.31 ± 0.07), nerve stability (GSD: 0.25 ± 0.06), affability (GSD: 0.37 ±0.08), and ability to cooperate (GSD: 0.28 ± 0.07; LR (0.35 ± 0.09) (33). The personality trait for shyness-boldness had heritabilities of 0.25 in the GSD and 0.27 in the Rottweiler on the Dog Mentality Assessment given at the SDTC (34). In a population of U.S. guide dogs, heritabilities for dog-directed aggression was 0.27 ± 0.12 and for non-social fear was 0.27 ± 0.09 in Golden Retrievers (GR) as assessed by the C-BARQ questionnaire [described in Duffy and Serpell (23)]. The C-BARQ trainability score had heritabilities of 0.46 ± 0.07 in LR, 0.47 ± 0.07 in GSD, and 0.20 ± 0.08 in Golden Retrievers (35). A study of a guide dog breeding program in the UK found heritabilities of 0.25 ± 0.09 for “Following when called” in LRs, GRs, and their crosses (36). As generations of genetic improvement accumulate, it will, almost certainly, be necessary to develop new selection criteria for traits and characteristics that are not part of the selection criteria chosen as most important at the onset.
- The application of these genetic principles to a large population of dogs such as the potential DDCoE program is more feasible and effective than their application to small working dog organizations or single private breeders (14, 37). In the past, these methods have been applied widely for the improvement of livestock species [e.g., milk yield in cattle (38), growth rate in beef cattle (39), and body weight in chickens (40)] and hip dysplasia in dogs (8).
- The production of crossbred dogs for scent detection may be considered as an option, especially if “market forces” indicate a need and a demand for utilizing crossbred dogs for odor detection. This option will be most viable by choosing purebreds as parents of the crosses that will yield crossbred females suitable for breeding. Crossbred males and females not chosen for breeding would be available for training as odor detection dogs, or for alternate career paths as determined by each dog's abilities.

### Research and development

- The DDCoE will develop and validate a quantitative scent detection aptitude test to determine whether a dog meets government contracting specifications. As a general measure of a dog's scent detecting ability, it will also be used as a phenotype for selective breeding decisions by the DDCoE.
- This aptitude test result could provide a basis for the release of dogs unlikely to be successful from the program, saving financial resources. Academic working dog centers are currently collecting phenotype data on pups as early as 8–12 weeks, so these tests can be validated.
- The decreasing costs of high-density whole-genome marker panels and even genome sequencing, along with the DDCoE's access to data on large numbers of dogs, can lead to progress in understanding the complex health and behavior traits in detector dogs (41). Genetic factors affecting complex traits such as behavior and hip joint conformation have been elusive because these traits are the result of genes at many loci along with environmental effects. Large data sets are required to find genes of small effect (42, 43).
- The use of genomic selection methods also depends on the availability of large data sets. In this method, breeding dogs are selected based on possession of sets of genetic markers contributing to variation in desired traits. These methods have been used with livestock species with large data sets available, but not with dogs.
- A refinement of our understanding of how puppy raising and training strategies influence success rates at older ages will be undertaken. Comparisons between different methods and environments, both within and between DDCoE member organizations, could facilitate this understanding. Using knowledge gained from these studies, it may be possible to create puppy rearing strategies that maximize the number of Federal agency acceptable puppies produced by a single strategy. This could enable the targeting of a specific Federal agency's needs with puppies reared by a particular strategy.
- At present, criteria defining what makes a dog acceptable for Federal government purchase differs among agencies (5). The DDCoE will work with the Federal government to standardize the definition of acceptance criteria for Federal government purchase across agencies, especially those that focus on purchasing dogs destined for odor detection.

### Education

- The DDCoE will be an educational center for the dissemination of knowledge obtained through breeding, selection, and raising of detection dogs.
- Educational programs will be directed at veterinarians, researchers, agencies, trainers, and handlers.

### Benchmarks of success

- Dog performance: an annual increase in the percentage of puppies enrolled in the program that are able to enter training based on a reduction of health issues and an improvement in puppy development.
- An annual increase in the percentage of dogs that enter the workforce.
- An annual increase in the average duration of working life.
- An annual reduction in the cost of producing the dogs.
- The research benchmark will be the collection of data from the stages of development and the analysis and application of the data that allows a target 10% annual improvement in genetics, training, and performance measures.

In summary, a comprehensive approach to increasing the availability and improving the overall quality of detection dogs is proposed. This approach incorporates experiences from working dog programs over the past several decades. Key components that set this program apart from early programs that no longer exist include (1) the cooperative non-governmental structure, (2) application of the most current genetic, reproductive, medical and behavioral knowledge to the breeding and raising of dogs, and (3) the ability to distribute dogs to a wide variety of end-users.

## Author contributions

CO, EL, EH, ST, and LW all provided content, edits, and review. All authors approved the manuscript.

### Conflict of interest statement

The authors declare that the research was conducted in the absence of any commercial or financial relationships that could be construed as a potential conflict of interest.
